# Rietveld refinement of langbeinite-type K_2_YHf(PO_4_)_3_

**DOI:** 10.1107/S1600536809027573

**Published:** 2009-07-18

**Authors:** Ivan V. Ogorodnyk, Igor V. Zatovsky, Nikolay S. Slobodyanik

**Affiliations:** a Department of Inorganic Chemistry, Taras Shevchenko National University, 64 Volodymyrska Str., 01601 Kyiv, Ukraine

## Abstract

Potassium yttrium hafnium tris­(orthophosphate) belongs to the langbeinite-family and is built up from [*M*O_6_] octa­hedra [in which the positions of the two independent *M* sites are mutually occupied by Y and Hf in a 0.605 (10):0.395 (10) ratio] and [PO_4_] tetra­hedra connected *via* vertices into a three-dimensional framework. This framework is penetrated by large closed cavities in which the two independent K atoms are located; one of the K atoms is nine-coordinated and the other is 12-coordinated by surrounding O atoms. The K, Y and Hf atoms lie on threefold rotation axes, whereas the P and O atoms are located in general positions.

## Related literature

For the structure of the mineral langbeinite, see: K_2_Mg_2_(SO_4_)_3_ (Zemann & Zemann, 1957 ▶). For powder diffraction investigations and Rietveld refinements of phosphate-based langbeinites, see: K_2_ *M*Zr(PO_4_)_3_, *M* = Y, Gd (Wulff *et al.*, 1992 ▶); K_2_FeZr(PO_4_)_3_ (Orlova *et al.*, 2003 ▶); K_2_ *Ln*Zr(PO_4_)_3_, *Ln* = Ce—Lu (Trubach *et al.*, 2004 ▶). Hafnium-containing phosphate langbeinites are reported for K_2_BiHf(PO_4_)_3_ (Losilla *et al.*, 1998 ▶) and K_1.93_Mn_0.53_Hf_1.47_(PO_4_)_3_ (Ogorodnyk *et al.*, 2007*a* ▶). For the synthesis of zirconium- or hafnium-containing langbeinite-related phosphates from fluoride precursors using flux techniques, see: Ogorodnyk *et al.* (2007*a* ▶,*b* ▶). Parameters needed to calculate bond-valence sums were taken from Brown & Altermatt (1985 ▶) and Brese & O’Keeffe (1991 ▶), respectively. For ionic radii, see: Shannon (1976 ▶). For crystallographic background, see: Boultif & Louër (2004 ▶).

## Experimental

### 

#### Crystal data

K_2_YHf(PO_4_)_3_
*M* *_r_* = 630.51
Cubic, 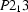

*a* = 10.30748 (9) Å
*V* = 1095.11 (2) Å^3^
*Z* = 4
Cu *K*α radiation
*T* = 293 K
Specimen shape: flat sheet
15 × 15 × 1 mm
Specimen prepared at 101.3 kPa
Specimen prepared at 293 K
Particle morphology: isometric, colourless

#### Data collection

Shimadzu XRD-6000 diffractometer
Specimen mounting: glass container
Specimen mounted in reflection mode
Scan method: step
2θ_min_ = 5.0, 2θ_max_ = 105.0°
Increment in 2θ = 0.02°

#### Refinement

*R*_p_ = 5.375
*R*_wp_ = 7.075
*R*_exp_ = 2.809
*R*_B_ = 4.248
*S* = 2.51
Wavelength of incident radiation: 1.540530 Å
Profile function: Thompson–Cox–Hastings pseudo-Voigt with axial divergence asymmetry (Thompson *et al.*, 1987 ▶)
528 reflections
48 parameters

### 

Data collection: *PCXRD* (Shimadzu, 2006 ▶); cell refinement: *FULLPROF* (Rodriguez-Carvajal, 2006 ▶); data reduction: *FULLPROF*; method used to solve structure: coordinates taken from an isotypic structure; program(s) used to refine structure: *FULLPROF*; molecular graphics: *DIAMOND* (Brandenburg, 1999 ▶); software used to prepare material for publication: *PLATON* (Spek, 2009 ▶) and *enCIFer* (Allen *et al.*, 2004 ▶).

## Supplementary Material

Crystal structure: contains datablocks global, I. DOI: 10.1107/S1600536809027573/wm2244sup1.cif

Rietveld powder data: contains datablocks I. DOI: 10.1107/S1600536809027573/wm2244Isup2.rtv

Additional supplementary materials:  crystallographic information; 3D view; checkCIF report

## Figures and Tables

**Table:** 

K1—O1^i^	2.981 (16)
K1—O2^ii^	3.345 (14)
K1—O4^ii^	3.413 (16)
K2—O3^ii^	2.907 (14)
K2—O2^iii^	2.912 (14)
K2—O4^ii^	3.207 (17)
K2—O4^iii^	3.336 (17)
*M*1—O1	2.148 (14)
*M*1—O2^iv^	2.085 (15)
*M*2—O3^i^	2.211 (14)
*M*2—O4	2.113 (17)
P1—O1	1.518 (16)
P1—O2	1.621 (17)
P1—O3	1.470 (16)
P1—O4	1.497 (19)

**Table:** 

O1—P1—O2	100.5 (9)
O1—P1—O3	113.0 (9)
O1—P1—O4	106.9 (9)
O2—P1—O3	121.4 (8)
O2—P1—O4	107.7 (9)
O3—P1—O4	106.5 (9)

Symmetry codes: (i) 
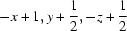
; (ii) 
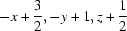
; (iii) 
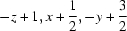
; (iv) 
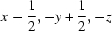
.

**Fig. 1.**
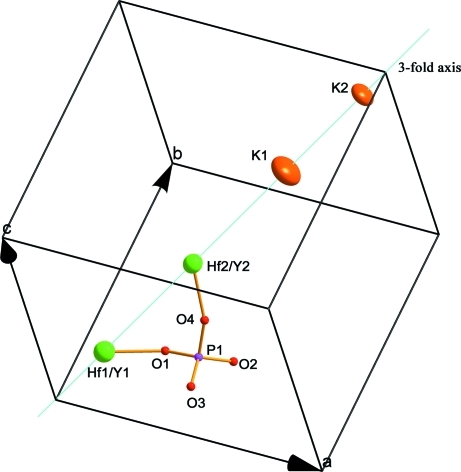
A view of the asymmetric unit of K2YHf(PO4)3. Displacement ellipsoids are drawn at the 50% probability level.

**Fig. 2.**
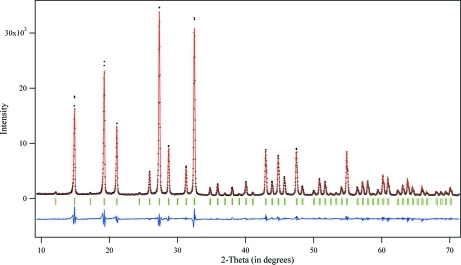
Rietveld refinement of K2YHf(PO4)3. Experimental (dots), calculated (red curve) and difference (blue curve) data for 2θ range 8-72°.

## supplementary crystallographic information

### Comment

Among a great variety of langbeinite-type based phosphate (mineral langbeinite K_2_Mg_2_(SO_4_)_3_, Zemann & Zemann, 1957), only several compounds containing hafnium were reported: K_2_BiHf(PO_4_)_3_ (Losilla *et al.*, 1998) and K_1.93_Mn_0.53_Hf_1.47_(PO_4_)_3_ (Ogorodnyk *et al.*, 2007*a*). At the same time a great number of zirconium-containing phosphates with langbeinite framework were synthesized and structurally characterized: K_2_*M*Zr(PO_4_)_3_, *M* = Y, Gd (Wulff *et al.*, 1992); K_2_FeZr(PO_4_)_3_ (Orlova *et al.*, 2003); K_2_LnZr(PO_4_)_3_, Ln = Ce—Lu (Trubach *et al.*, 2004*b*); Cs_1+_*_x_*Ln*_x_*Zr_2-_*_x_*(PO_4_)_3_, Ln = Sm—Lu (Ogorodnyk *et al.*, 2007*b*). This can be connected with the similarity of chemical behavior of zirconium and hafnium on the one hand and the rareness (or, possibly, the high prices) of hafnium raw materials in comparison with zirconium ones on the other hand.

Due to similar chemical properties of Zr and Hf and the close values of their ionic radii (for coordination number 6 they are 0.72 and 0.71 Å for Zr and Hf, respectively; Shannon, 1976) the cell parameters of K_2_YHf(PO_4_)_3_ are slightly smaller than of K_2_YZr(PO_4_)_3_ (*a*= 10.3346 (1) Å; Wulff *et al.*, 1992).

K, Y and Hf atoms lie on the 3-fold rotation axes in 4*a* positions (Fig. 1). P and O atoms are located in 12*b* positions. Both Y and Hf atoms occupy two hexacoordinated positions competitively. *M*1 position is preferably occupied by Hf while *M*2 is by Y. The structure contains [*M*O_6_] octahedra and [PO_4_] tetrahedra which are connected *via* vertices. Two nearest [*M*O_6_] octahedra are joined to each other by three bridging orthophosphate tetrahedra forming {*M*_2_P_3_O_18_} groups. These groups form three-dimensional framework penetrated with large closed cavities. Two independent potassium atoms are located in each cavity. K1 atom is nine-coordinated, while K2 is twelve-coordinated.

Bond valence sums (BVS) were calculated using parameters for Hf, Y, P from Brese & O'Keeffe (1991) and for K from Brown & Altermatt (1985). The calculation were performed for formula sum K_2_YHf(PO_4_)_3_ taking into account occupancies of the octahedrally coordinatedd *M* positions. The sum of BVS of positively charged atoms is equal to 24.16 while the chemical charge of the remaining O atoms is equal to -24.

### Experimental

Well-shaped tetrahedral crystals of K_2_YHf(PO_4_)_3_ were grown using a flux technique. A mixture of 4.52 g KPO_3_ and 3.4 g K_4_P_2_O_7_ (initial K/P molar ratio was set equal to 1.35) was melted in a platinum crucible at 1273 K. The melt was kept at this temperature for 1 h and after that the temperature was decreased to 1173 K. Dispersed in an agate mortar, a mixture of 1.36 g HfF_4_ and 0,78 g YF_3_ was added to the phosphate flux under stirring. The crystallization of the melts was performed from 1173 to 893 K at a rate of 30 K/h. The synthesized crystalline sample was separated from remaining glass by leaching with hot water. The dimensions of the crystals were found to be in a range 0.01–0.05 mm. The sample was ground in an agate mortar before performing powder XRD data collection. The recorded powder pattern indicated a single phase material.

The element ratio was determined using ICP-AES analyses (Shimadzu ICPE-9000 spectrometer). The sample for measurements was prepared by dissolution of calculated amount of K_2_YHf(PO_4_)_3_ in sulfuric acid (98%) with final dilution by bidistilled water. Element ratio was found to be: 2.02:0.97:0.98:3.04 for K:Y:Hf:*P* which fits well with the theoretical values.

### Refinement

The cubic cell was found by Dicvol 2004 (Boultif & Louër, 2004). The Hf-containing langbeinite-related compound with general composition K_1.93_Mn_0.53_Hf_1.47_(PO_4_)_3_ (ICSD-418669, Ogorodnyk *et al.*, 2007*a*) was selected as a starting model for Rietveld refinement. At first profile matching refinement was performed. Then background and scaling factors were added to the refined parameters. The background was approximated using a 6-coefficient polynomial function. Modified pseudo-Voigt function (Thompson *et al.*, 1987) was used for the profile refinement. On the next stage atomic positions were refined. Due to previous investigations of langbeinite-related phosphates and close ionic radii of Y and Hf common positions *M*1 and *M*2 occupied by both these elements were suggested. Their coordinates, anisotropic displacement parameters (ADP) and occupancies were constrained. After the refinement of the metal occupancies in *M*1 and *M*2 positions and refinement of isotropic displacement parameters, we tried to refine ADPs of the heavy atoms (Y, Hf and K). The isotropic displacement parameters of the four O atoms were constrained to be equal before the final cycles of the refinement. The experimental, calculated and difference pattern of the Rietveld refinement is shown in Fig. 2.

### Crystal data

**Table:**

K_2_HfY(PO_4_)_3_	Cu *K*α radiation, λ = 1.540530 Å
*M**_r_* = 630.51	*T* = 293 K
Cubic, *P*2_1_3	Particle morphology: isometric
*a* = 10.30748 (9) Å	colourless
*V* = 1095.11 (2) Å^3^	flat_sheet, 15 × 15 mm
*Z* = 4	Specimen preparation: Prepared at 293 K and 101.3 kPa
*D*_x_ = 3.824 Mg m^−^^3^	

### Data collection

**Table:**

Shimadzu XRD-6000 diffractometer	Data collection mode: reflection
Radiation source: X-ray tube, X-ray	Scan method: step
graphite	2θ_min_ = 5.02°, 2θ_max_ = 105.02°, 2θ_step_ = 0.02°
Specimen mounting: glass container	

### Refinement

**Table:**

*R*_p_ = 5.375	Profile function: Thompson–Cox–Hastings pseudo-Voigt Axial divergence asymmetry (Thompson *et al.*, 1987)
*R*_wp_ = 7.075	48 parameters
*R*_exp_ = 2.809	0 restraints
*R*_Bragg_ = 4.248	14 constraints
*R*(*F*) = 3.14	Standard least squares refinement
χ^2^ = 6.300	(Δ/σ)_max_ = 0.001
5001 data points	Background function: FullProf Background 6-coeficient polynomial function
Excluded region(s): undef	

### Special details

**Table:**

Geometry. Bond distances, angles *etc*. have been calculated using the rounded fractional coordinates. All su's are estimated from the variances of the (full) variance-covariance matrix. The cell e.s.d.'s are taken into account in the estimation of distances, angles and torsion angles

### Fractional atomic coordinates and isotropic or equivalent isotropic displacement parameters (Å^2^)

**Table:**

	*x*	*y*	*z*	*U*_iso_*/*U*_eq_	Occ. (<1)
K1	0.6984 (4)	0.6984 (4)	0.6984 (4)	0.12 (9)	
K2	0.9307 (5)	0.9307 (5)	0.9307 (5)	0.07 (9)	
Y1	0.14694 (11)	0.14694 (11)	0.14694 (11)	0.06 (9)	0.395 (10)
Y2	0.41559 (18)	0.41559 (18)	0.41559 (18)	0.05 (9)	0.605 (10)
Hf1	0.14694 (11)	0.14694 (11)	0.14694 (11)	0.06 (9)	0.605 (10)
Hf2	0.41559 (18)	0.41559 (18)	0.41559 (18)	0.05 (9)	0.395 (10)
P1	0.4609 (5)	0.2311 (8)	0.1292 (8)	0.06 (9)	
O1	0.3207 (14)	0.2448 (13)	0.0864 (15)	0.07 (9)	
O2	0.5337 (12)	0.3118 (13)	0.0155 (16)	0.07 (9)	
O3	0.4970 (13)	0.0965 (13)	0.1595 (13)	0.07 (9)	
O4	0.4750 (14)	0.3063 (15)	0.2526 (17)	0.07 (9)	

### Atomic displacement parameters (Å^2^)

**Table:**

	*U*^11^	*U*^22^	*U*^33^	*U*^12^	*U*^13^	*U*^23^
K1	0.12 (9)	0.12 (9)	0.12 (9)	−0.031 (4)	−0.031 (4)	−0.031 (4)
K2	0.07 (9)	0.07 (9)	0.07 (9)	−0.019 (3)	−0.019 (3)	−0.019 (3)
Y1	0.06 (9)	0.06 (9)	0.06 (9)	0.0018 (9)	0.0018 (9)	0.0018 (9)
Y2	0.05 (9)	0.05 (9)	0.05 (9)	0.0034 (9)	0.0034 (9)	0.0034 (9)
Hf1	0.06 (9)	0.06 (9)	0.06 (9)	0.0018 (9)	0.0018 (9)	0.0018 (9)
Hf2	0.05 (9)	0.05 (9)	0.05 (9)	0.0034 (9)	0.0034 (9)	0.0034 (9)

### Geometric parameters (Å, °)

**Table:**

K1—O1^i^	2.981 (16)	Hf1—O1^xiii^	2.148 (14)
K1—O2^ii^	3.345 (14)	Hf1—O2^xiv^	2.085 (15)
K1—O4^ii^	3.413 (16)	Hf2—O3^i^	2.211 (14)
K1—O1^iii^	2.981 (16)	Hf2—O4	2.113 (17)
K1—O2^iv^	3.345 (14)	Hf2—O4^xi^	2.113 (17)
K1—O4^iv^	3.413 (16)	Hf2—O3^iii^	2.211 (14)
K1—O1^v^	2.981 (16)	Hf2—O4^xiii^	2.113 (17)
K1—O2^vi^	3.345 (14)	Hf2—O3^v^	2.211 (14)
K1—O4^vi^	3.413 (16)	Y1—O1	2.148 (14)
K2—O3^ii^	2.907 (14)	Y1—O2^x^	2.085 (15)
K2—O2^vii^	2.912 (14)	Y1—O2^xii^	2.085 (15)
K2—O4^ii^	3.207 (17)	Y1—O1^xiii^	2.148 (14)
K2—O4^vii^	3.336 (17)	Y1—O2^xiv^	2.085 (15)
K2—O3^iv^	2.907 (14)	Y1—O1^xi^	2.148 (14)
K2—O2^viii^	2.912 (14)	Y2—O3^i^	2.211 (14)
K2—O4^iv^	3.207 (17)	Y2—O4	2.113 (17)
K2—O4^viii^	3.336 (17)	Y2—O3^v^	2.211 (14)
K2—O3^vi^	2.907 (14)	Y2—O4^xi^	2.113 (17)
K2—O2^ix^	2.912 (14)	Y2—O3^iii^	2.211 (14)
K2—O4^vi^	3.207 (17)	Y2—O4^xiii^	2.113 (17)
K2—O4^ix^	3.336 (17)	P1—O1	1.518 (16)
Hf1—O1	2.148 (14)	P1—O2	1.621 (17)
Hf1—O2^x^	2.085 (15)	P1—O3	1.470 (16)
Hf1—O1^xi^	2.148 (14)	P1—O4	1.497 (19)
Hf1—O2^xii^	2.085 (15)		
			
O1—Hf1—O2^x^	97.9 (5)	O2^x^—Y1—O2^xii^	80.1 (5)
O1—Hf1—O1^xi^	89.3 (5)	O1^xiii^—Y1—O2^x^	172.6 (5)
O1—Hf1—O2^xii^	172.6 (5)	O2^x^—Y1—O2^xiv^	80.1 (5)
O1—Hf1—O1^xiii^	89.3 (5)	O1^xi^—Y1—O2^xii^	97.9 (5)
O1—Hf1—O2^xiv^	92.5 (5)	O1^xi^—Y1—O1^xiii^	89.3 (5)
O1^xi^—Hf1—O2^x^	92.5 (5)	O1^xi^—Y1—O2^xiv^	172.6 (5)
O2^x^—Hf1—O2^xii^	80.1 (5)	O1^xiii^—Y1—O2^xii^	92.5 (5)
O1^xiii^—Hf1—O2^x^	172.6 (5)	O2^xii^—Y1—O2^xiv^	80.1 (5)
O2^x^—Hf1—O2^xiv^	80.1 (5)	O1^xiii^—Y1—O2^xiv^	97.9 (5)
O1^xi^—Hf1—O2^xii^	97.9 (5)	O3^i^—Y2—O4	93.1 (6)
O1^xi^—Hf1—O1^xiii^	89.3 (5)	O4—Y2—O4^xi^	87.8 (6)
O1^xi^—Hf1—O2^xiv^	172.6 (5)	O3^iii^—Y2—O4	171.5 (6)
O1^xiii^—Hf1—O2^xii^	92.5 (5)	O4—Y2—O4^xiii^	87.8 (6)
O2^xii^—Hf1—O2^xiv^	80.1 (5)	O3^v^—Y2—O4	83.9 (5)
O1^xiii^—Hf1—O2^xiv^	97.9 (5)	O3^i^—Y2—O4^xi^	83.9 (5)
O3^i^—Hf2—O4	93.1 (6)	O3^i^—Y2—O3^iii^	95.4 (5)
O4—Hf2—O4^xi^	87.8 (6)	O3^i^—Y2—O4^xiii^	171.5 (6)
O3^iii^—Hf2—O4	171.5 (6)	O3^i^—Y2—O3^v^	95.4 (5)
O4—Hf2—O4^xiii^	87.8 (6)	O3^iii^—Y2—O4^xi^	93.1 (6)
O3^v^—Hf2—O4	83.9 (5)	O4^xi^—Y2—O4^xiii^	87.8 (6)
O3^i^—Hf2—O4^xi^	83.9 (5)	O3^v^—Y2—O4^xi^	171.5 (6)
O3^i^—Hf2—O3^iii^	95.4 (5)	O3^iii^—Y2—O4^xiii^	83.9 (5)
O3^i^—Hf2—O4^xiii^	171.5 (6)	O3^iii^—Y2—O3^v^	95.4 (5)
O3^i^—Hf2—O3^v^	95.4 (5)	O3^v^—Y2—O4^xiii^	93.1 (6)
O3^iii^—Hf2—O4^xi^	93.1 (6)	O1—P1—O2	100.5 (9)
O4^xi^—Hf2—O4^xiii^	87.8 (6)	O1—P1—O3	113.0 (9)
O3^v^—Hf2—O4^xi^	171.5 (6)	O1—P1—O4	106.9 (9)
O3^iii^—Hf2—O4^xiii^	83.9 (5)	O2—P1—O3	121.4 (8)
O3^iii^—Hf2—O3^v^	95.4 (5)	O2—P1—O4	107.7 (9)
O3^v^—Hf2—O4^xiii^	93.1 (6)	O3—P1—O4	106.5 (9)
O1—Y1—O2^x^	97.9 (5)	Hf1—O1—P1	131.7 (9)
O1—Y1—O1^xi^	89.3 (5)	Y1—O1—P1	131.7 (9)
O1—Y1—O2^xii^	172.6 (5)	Hf1^xv^—O2—P1	160.8 (9)
O1—Y1—O1^xiii^	89.3 (5)	Hf2^xvi^—O3—P1	145.6 (9)
O1—Y1—O2^xiv^	92.5 (5)	Hf2—O4—P1	157.5 (10)
O1^xi^—Y1—O2^x^	92.5 (5)	Y2—O4—P1	157.5 (10)

Symmetry codes: (i) −*x*+1, *y*+1/2, −*z*+1/2; (ii) −*x*+3/2, −*y*+1, *z*+1/2; (iii) −*z*+1/2, −*x*+1, *y*+1/2; (iv) −*y*+1, *z*+1/2, −*x*+3/2; (v) *y*+1/2, −*z*+1/2, −*x*+1; (vi) *z*+1/2, −*x*+3/2, −*y*+1; (vii) −*z*+1, *x*+1/2, −*y*+3/2; (viii) −*y*+3/2, −*z*+1, *x*+1/2; (ix) *x*+1/2, −*y*+3/2, −*z*+1; (x) *x*−1/2, −*y*+1/2, −*z*; (xi) *z*, *x*, *y*; (xii) −*z*, *x*−1/2, −*y*+1/2; (xiii) *y*, *z*, *x*; (xiv) −*y*+1/2, −*z*, *x*−1/2; (xv) *x*+1/2, −*y*+1/2, −*z*; (xvi) −*x*+1, *y*−1/2, −*z*+1/2.
